# 50% effective concentration of sevoflurane for immobility in cerebral palsy children undergoing botulinum toxin injection

**DOI:** 10.1097/MD.0000000000030928

**Published:** 2022-10-21

**Authors:** Kanghui Kim, Eunhee Lee, Sung Mee Jung, Jongyoon Baek

**Affiliations:** a Department of Anesthesiology and Pain Medicine, Yeungnam University College of Medicine, Daegu, Republic of Korea.

**Keywords:** botulinum toxin type A, cerebral palsy, deep sedation, sevoflurane

## Abstract

This prospective study aimed to determine the optimum end-tidal sevoflurane concentration required for immobility during botulinum toxin injection in spontaneously breathing children with cerebral palsy (CP).

**Methods:**

Twenty-three children with spastic CP, aged 3 to 12 years, with American Society of Anesthesiologists (ASA) physical status I and II, scheduled to receive botulinum toxin type A injection were enrolled in the study. After induction of deep sedation using pre-filled 8% sevoflurane in oxygen and maintenance of the predetermined end-tidal sevoflurane concentration, the botulinum toxin was injected in spontaneously breathing children. The response to the botulinum toxin injection was classified as “movement” or “no movement” by an independent investigator who was blinded to the predetermined end-tidal sevoflurane concentration and bispectral index (BIS) value. The end-tidal sevoflurane concentration was predetermined, initiating at 2.0% with 0.2% as a step size in the next patient depending on the previous patient’s response using the modified Dixon’s up-and-down method.

**Results:**

Of 21 children, 12 (57.1%) showed “no movement” in response to the botulinum toxin injection. By Dixon’s up-and-down method, the 50% effective end-tidal concentration (EC_50_) of sevoflurane for successful botulinum toxin injection was 1.76 ± 0.15% (95% CI 1.62–1.90). Based on the dose‐response curve using probit analysis, the predicted EC_50_ and 95% effective end-tidal concentrations (EC_95_) of sevoflurane without movement were 1.77% (95% CI 1.59–2.35) and 2.09% (95% CI 1.89–5.80), respectively.

**Conclusion:**

Botulinum toxin injection can be successfully accomplished at an end-tidal sevoflurane concentration of 1.76 ± 0.15% in 50% of spontaneously breathing children with CP aged 3–12 years.

## 1. Introduction

Cerebral palsy (CP) is the most common cause of spastic movement disorders in children.^[1]^ With ongoing child motor development, spasticity associated with contraction, clonus, and hyperreflexia leads to fixed contracture and progressive musculoskeletal deformity, such as impairment of function and reduced muscle growth. Although several management strategies for spasticity have been introduced in the last several decades,^[2]^ intramuscular injection of botulinum toxin type A for focal spasticity has been established as an important part of multimodal management to support motor development and improve posture and function, relieve pain, and ease care in children with CP.^[3]^ The correct needle placement in the target muscle is essential for the optimal efficacy and safety of botulinum toxin injection. Therefore, effective analgesia and sedation are frequently required to maintain immobility in children with CP who are unable to control movement against pain and anxiety during botulinum toxin injection.

Although diverse pharmacological and non-pharmacological sedation protocols to offer immobility for botulinum toxin injection have been used in clinical practice,^[4,5]^ there are insufficient data to support the superiority of any modality. Selecting the lowest dose of the drug with the highest therapeutic index for the procedure is essential for adequate and safe sedation. The use of inhaled anesthetic agents enables target-controlled titration according to the sedation level through real-time objective measurement of end-tidal concentrations. In addition, inhaled anesthetics suppress both sensory processing of noxious stimuli and spinal motor neuron excitability, thus providing immobility in the setting of noxious stimuli.^[6]^

Sevoflurane has been commonly used for pediatric inhaled sedation with distinct advantages of rapid onset and recovery, precise control of anesthetic depth, wide range of safety profile, and preservation of spontaneous ventilation even with deep sedation.^[7–9]^ In addition, the lack of pungency and minimal odor make sevoflurane an excellent candidate for mask induction. However, most data regarding the minimum alveolar concentration (MAC) of sevoflurane for deep sedation come from its use in healthy children. Despite the clinical utility of sevoflurane deep sedation in children with CP,^[10]^ the end-tidal sevoflurane concentration for immobility during painful procedures such as the administration of botulinum toxin injection has not yet been established.

Therefore, in this prospective study, we aimed to determine the optimum end-tidal concentration of sevoflurane required for immobility in response to botulinum toxin type A injection in children with spastic CP.

## 2. Methods

This prospective study protocol was approved by the Institutional Review Board (YUMC-2018-04-023) and registered at ClinicalTrials.gov (NCT03553446) and CRiS (KCT0007372). Written informed consent from the participants’ parents or guardians and, when appropriate, verbal assent from the participating child were obtained before enrollment in the study. Twenty-three children with spastic CP aged between 3 and 12 years, American Society of Anesthesiologists (ASA) physical status I and II, who were scheduled for botulinum toxin injection under deep sedation were consecutively enrolled in the study. All participants had previously failed to receive their botulinum toxin injection under conscious sedation outside the operating room. Patients with anticipated difficult airway, unstable cardiac disease, craniofacial defects, allergy to drugs used in this study, family history of malignant hyperthermia, recent (<8 weeks) history of pneumonia, bronchitis, asthma, or upper respiratory infection, difficulty in applying mask induction, and ASA physical status > II were excluded from the study.

A peripheral 22/24 gauge intravenous catheter was inserted by an expert nurse 1 hour before the estimated procedure as per hospital standard practice and as an alternative route of intravenous anesthetics in situations where inhalational induction is difficult due to the unexpected patient refusal or withdrawal of consent. None of the children were taking premedication. After patient arrival to the operating room, accompanied with one of the parents or a guardian, continuous monitoring of oxygen saturation by pulse oximetry, heart rate by electrocardiography and noninvasive blood pressure (NIBP) measurement every 5 minutes were performed in the supine position. The depth of sedation was also monitored using the bispectral index (BIS, VISTA^TM^; Aspect Medical System, Newton, MA) with an age- and head size-appropriate BIS sensor. All baseline data were collected before sevoflurane administration. An independent investigator, who was blinded to predetermined end-tidal sevoflurane concentration, assessed the level of sedation using clinical sedation scales, such as the University of Michigan Sedation Scale (UMSS: 0 = awake and alert, 1 = tired/sleepy, appropriate response to verbal conversation and/or sound, 2 = somnolent/sleeping, easily aroused with a simple verbal commend or light tactile stimulation, 3 = deep sleep, arousable only with a significant physical stimulation, 4 = unarousable) and Modified Observer’s Assessment of Alertness and Sedation scale (MOAAS: 0 = no response after painful trapezius squeeze, 1 = responds only after painful trapezius squeeze, 2 = responds only after mild prodding or shaking, 3 = responds only after name is called loudly and/or repeatedly, 4 = lethargic response to name spoken in normal tone, 5 = responds readily to name spoken in normal tone) and BIS values at loss of consciousness, localization using nerve stimulator, needle placement, botulinum toxin injection, end of procedure, and eye opening to verbal command.

After prefilling the semi-closed circuit system (Datex-Ohmeda S/5 monitor; Datex-Ohmeda, Madison, WI), each patient received 8% sevoflurane in oxygen at a fresh gas flow rate of 5 L/min via a tight-fitted face mask for induction of deep sedation with preservation of spontaneous breathing. When the child did not respond to verbal commands three times, confirmed that there was no gas leakage by securing the mask to the face with a mask harness and then the sevoflurane dial setting was adjusted to achieve a predetermined target end-tidal sevoflurane concentration. The inspired and end-tidal concentration of CO_2_ partial pressure (etCO_2_) and sevoflurane were continuously measured from a side-stream sampling cannula located at the elbow of breathing circuit using a gas monitor connected to an anesthetic machine. The respiratory gas analyzer was calibrated using a standard gas mixture before the induction of sedation. During the study, the mask fixed on the patient’s face was carefully checked to prevent gas leakage such as a decrease in etCO_2_ and end-tidal sevoflurane concentration due to the patient movement. Tidal volume and respiratory rate, which can affect the measurement of end-tidal sevoflurane concentration were closely monitored. Once inspired and end-tidal sevoflurane concentration equilibrated at a predetermined target concentration, these settings were maintained for 6 minutes to equilibrate sevoflurane concentration between the blood and brain.^[11,12]^ The child was then positioned, and localization of the target muscle using a nerve stimulator for botulinum toxin injection was attempted.

The modified Dixon’s up-and-down sequential allocation method was used to determine the end-tidal sevoflurane concentration for the next child.^[13]^ The first child received an end-tidal sevoflurane concentration of 2.0%, which was the expected minimum end-tidal concentration of sevoflurane for successful completion of the painful procedure. At the target sevoflurane concentration, the response to botulinum toxin injection in the child was classified as either “movement” or “no movement” by another investigator who was blinded to the sevoflurane concentration and BIS value. If the child showed movement such as purposeful withdrawal of limbs to botulinum toxin injection, the target end-tidal sevoflurane concentration for the next child was increased by 0.2%. In contrast, if the child did not show movement, the target end-tidal sevoflurane concentration for the next child was reduced by 0.2%. The end-tidal sevoflurane concentration was increased in children showing movement until immobility was achieved. Considering the starting concentration, increment size and interindividual variability^[14,15]^ in CP children aged between 3 and 12 years old, the process of assessing movement was continued until at least seven crossover points were obtained to decrease the likelihood of reporting an inaccurate estimate.^[16]^ The corresponding end-tidal concentration of sevoflurane to a mid-point between “movement” and “ no movement” was defined as 50% effective end-tidal concentration (EC_50_) of sevoflurane for one crossover. Any adverse events, such as hypoxemia (oxygen saturation < 93%), hypercapnia (etCO_2_ > 50 mm Hg), apnea (>20 s), and 20% reduction in the heart rate or NIBP from the baseline values, were recorded during sedation. During the post-anesthesia care unit (PACU) stay, delirium (using the pediatric anesthesia emergence delirium [PAED] scale), nausea, vomiting, headache, and intraoperative awareness were also observed.

The sample size was calculated based on a previous study in which the study was terminated after obtaining seven crossovers.^[16]^ Statistical analysis was performed using IBM SPSS version 22.0 for Windows (IBM Corp., Armonk, NY). Continuous variables are presented as median (interquartile range) or mean and standard deviation (SD), and categorical variables are presented as number of patients (%). Hemodynamic, respiratory, and sedative data over time were analyzed using repeated measures of analysis of variance. The EC_50_ of sevoflurane required for immobility during botulinum toxin injection was determined as the mean value of seven independent crossover points of consecutive subjects between “no movement” and “movement” using modified Dixon’s up-and-down method. The predicted EC_50_ and 95% effective end-tidal concentration (EC_95_) (with 95% confidence interval [CI]) of sevoflurane for botulinum toxin injection were obtained by a dose‐response curve using probit regression analysis. Statistical significance was set at *P* < .05.

## 3. Results

**Figure 1.**
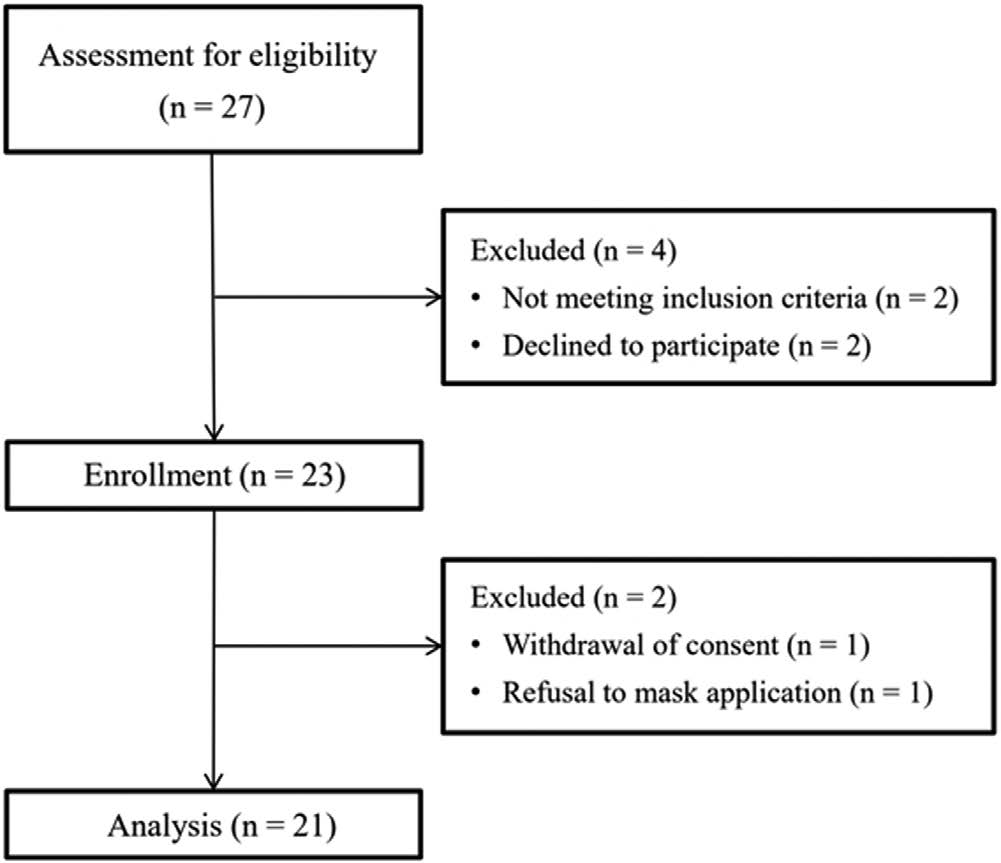
Flow diagram.

Of the 27 children with spastic CP assessed for study eligibility, 23 met the inclusion criteria (Fig. 1). After enrollment, two patients were excluded due to withdrawal of consent and difficulty in applying mask ventilation, and a total of 21 children completed the study. Detailed patient characteristics are shown in Table 1. Nine (42.9%) patients at each target end-tidal concentration of sevoflurane showed purposeful limb movement in response to botulinum toxin injection.

**Table 1:** Patient characteristics.

Parameter	
Age (yr)	5.0 (4.5–7.5)
Male (n)	15 (71.4)
Body mass index (kg/m^2^)	16.9 ± 2.9
Coexisting disease (n)	
Mental retardation	1 (4.8)
GMFCS (n)	
I/II/III	1 (4.8)/8 (38.1)/5 (23.8)
IV/V	5 (23.8)/2 (9.5)
Type of spasticity (n)	
Diplegia/hemiplegia/quadriplegia	18 (85.7)/1 (4.8)/2 (9.5)
Baseline BIS value	94.8 ± 4.4

Values are presented as median (interquartile ranges) or mean ± standard deviation for continuous variables and number of patients (%) for categorical variables.

BIS = bispectral index, GMFCS = gross motor function classification system.

**Figure 2.**
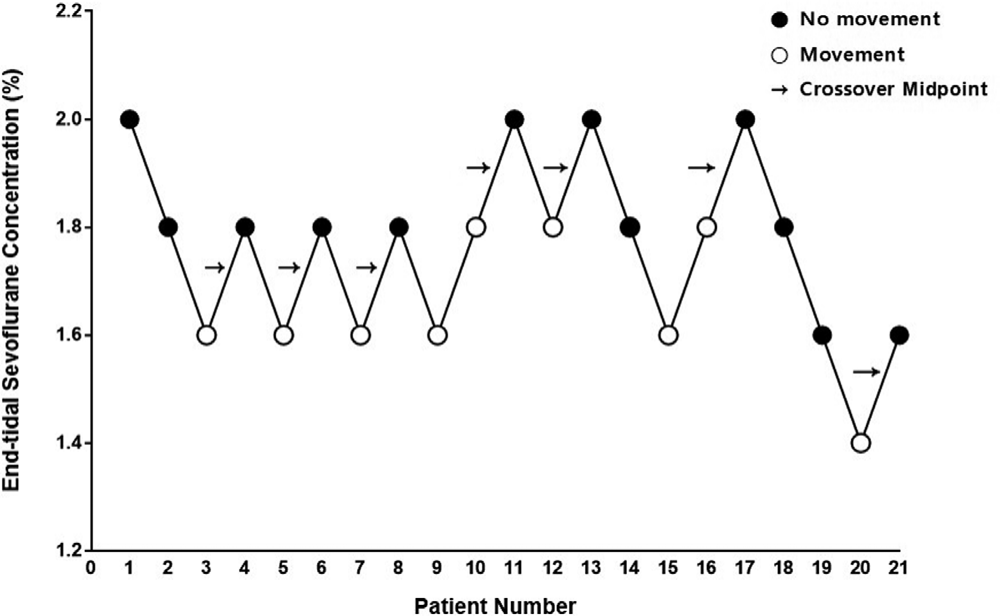
Response to botulinum toxin injection at predetermined end-tidal concentration of sevoflurane. The predetermined end-tidal sevoflurane concentration started at 2.0% with a step size of 0.2% using modified Dixon’s up-and-down method. Arrows indicate the midpoint concentration of all independent crossover pairs from “movement” to “no movement” in 21 consecutive children with spastic cerebral palsy.

**Figure 3.**
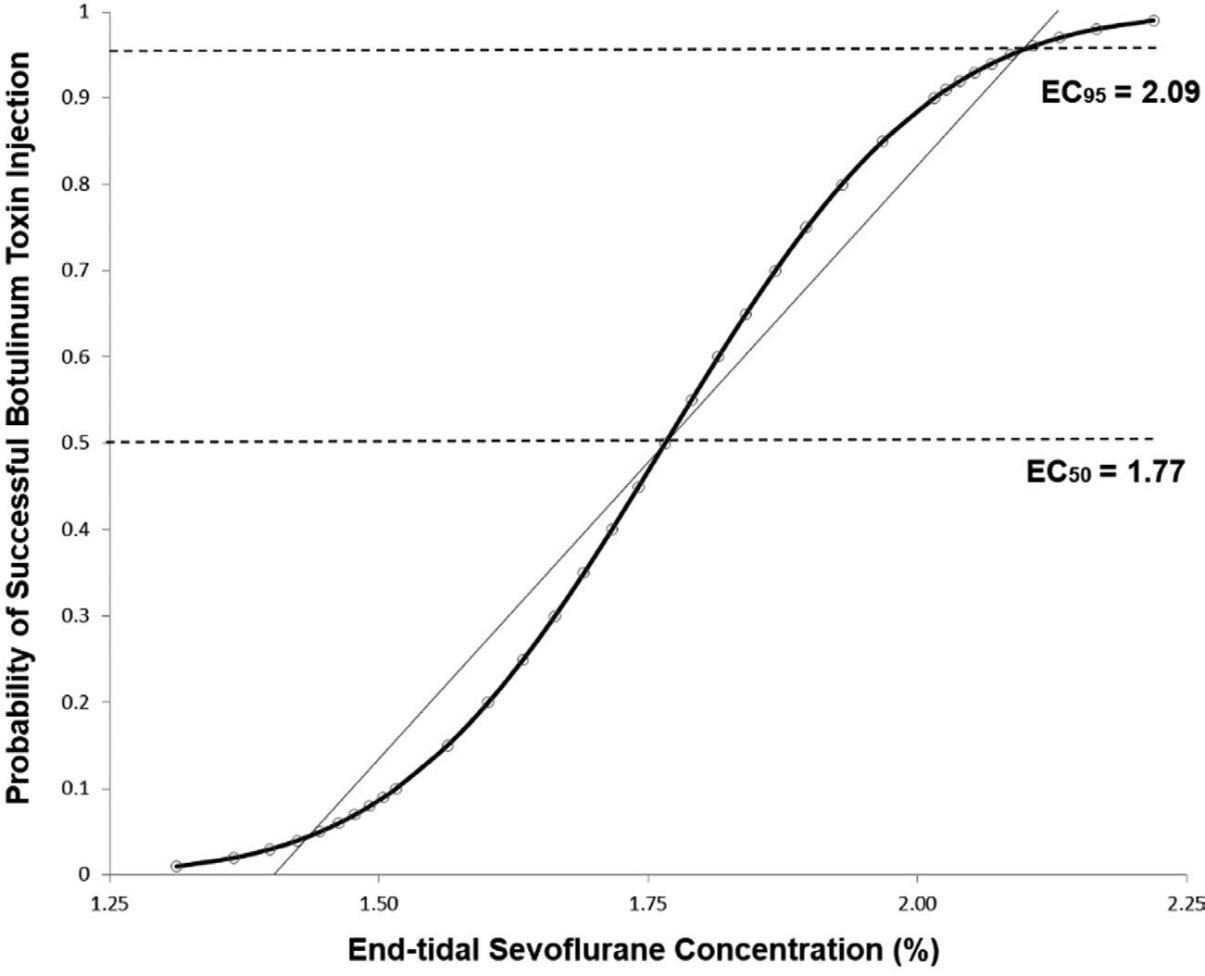
Dose response curve plotted from the probit analysis.

Using Dixon’s up-and-down method, the EC_50_ of sevoflurane for successful botulinum toxin injection in spontaneously breathing children with CP was 1.76 ± 0.15% (95% CI 1.62–1.90) (Fig. 2). By probit regression analysis, the predicted EC_50_ and EC_95_ of sevoflurane for successful botulinum toxin injection in children with CP were 1.77% (95% CI 1.59–2.35) and 2.09% (95% CI 1.89–5.80), respectively (Fig. 3). Changes in hemodynamic and respiratory data, BIS values, and UMSS and MOAAS scores throughout the study period in patients with “movement” and “no movement” were comparable. However, the UMSS scores [3.8 ± 0.3 vs 3.0 ± 0.5, *P* = .001] were higher and MOAAS scores [0.3 ± 0.4 vs 1.1 ± 0.3, *P* < .001] were lower in patients with “movement” than in those without movement at needle placement.

Apnea and subsequent transient desaturation were observed in 1 (4.8%) patient at the measured end-tidal sevoflurane concentration of 1.8% during the procedure, but the patient soon recovered spontaneous ventilation with jaw thrust and temporary positive pressure ventilation and showed movement during the procedure (Table 2). During the stay at the PACU, 1 (4.8%) and 2 (9.5%) patients complained of headache and nausea, respectively. However, their symptoms were relieved without medication before discharge from the PACU.

**Table 2:** Intraoperative and postoperative data.

Parameter	
Position (n)	
Supine/prone/both	1 (4.8)/17 (80.9)/3 (14.3)
Duration	
Induction (s)	30 (17.5–43.5)
Sedation (min)	27.8 ± 8.0
Procedure (min)	17.9 ± 7.4
Emergence (min)	6 (5.0–7.0)
BIS value at awakening	79.7 ± 9.4
PAED score	7.0 (6.0–8.0)
Adverse event (n)	
During sedation	
Hypoxia (SpO_2_ < 93%)	1 (4.8)
Apnea (>20 s)	1 (4.8)
During PACU stay	
Nausea	2 (9.5)
Headache	1 (4.8)

Values are presented as median (interquartile range) or mean ± standard deviation for continuous variables and number of patients (%) for categorical variables.

BIS = bispectral index, Emergence = duration from discontinuation of sevoflurane inhalation to eye opening to verbal command, PACU = post-anesthesia care unit, PAED = pediatric anesthesia emergence delirium.

## 4. Discussion

The main finding of our study is that botulinum toxin injection without movement was accomplished at 1.76 ± 0.15% of the end-tidal sevoflurane concentration in 50% of children with CP aged between 3 and 12 years with preservation of spontaneous breathing. The predicted EC_50_ and EC_95_ of sevoflurane were 1.77% and 2.09%, respectively, by a dose‐response curve using probit regression analysis. In addition, we found that sevoflurane deep sedation provided acceptable immobility for botulinum toxin injection with minimal adverse respiratory events. To our knowledge, this is the first study to determine the optimum end-tidal concentration of sevoflurane for botulinum toxin injection in children with CP.

In contrast to other inhaled anesthetics, the MAC of sevoflurane does not decrease steadily as age increases in the pediatric population. The MAC of sevoflurane is 3.2% to 3.3% in neonates and infants younger than 6 months of age and is 2.5% to 2.6% without change in older infants and children up to 12 years of age in normal children.^[17]^ Therefore, we enrolled children aged between 3 and 12 years. Until now, only one retrospective study reported that an inspired sevoflurane concentration of 2.5% to 4.0% was effective and safe for botulinum toxin injection in children with CP.^[10]^ However, authors did not report the end-tidal sevoflurane concentration. We assumed that the sevoflurane concentration required for botulinum toxin injection would be lower than sevoflurane MAC estimated in children without CP because children with CP have higher sensitivity to sevoflurane^[18–20]^ and intramuscular injection using a 25 gauge needle may result in less intensity of stimulation compared with the skin incision. The EC_50_ of sevoflurane (1.76%) for successful injection of botulinum toxin in children with CP in the present study is lower than those (approximately 2.03–2.50%) reported in children without CP with similar age ranges for skin incision.^[17,21]^

In the present study, the BIS values during deep sedation were maintained between 50 and 60 and were not different between children showing “movement” and those without “movement.” This suggests that end-tidal sevoflurane concentration required to achieve immobility in response to a noxious stimulus is higher than that required to produce hypnosis. Moreover, it appears that BIS is not reliable in predicting immobility to botulinum toxin injection when using sevoflurane for deep sedation. Therefore, careful titration of the end-tidal sevoflurane concentration combined with proper assessment of sedation status based on the response to stimulation would be needed to achieve deep sedation with immobility in this painful procedure in children with CP.

It is considered that children with CP may present physical characteristics and comorbidities that put them at greater risk of adverse events during general anesthesia.^[22]^ However, in spite of the potential risk of easy progression into a level of general anesthesia, the overall incidence of adverse events was minimal, the most frequent being nausea and vomiting (3.88%) followed by transient hypoxemia (2.07%), in deep sedated children with CP using sevoflurane for botulinum toxin injection.^[10]^ We also observed apnea-related hypoxemia during sedation in 4.8% and nausea after emergence in 9.5% of patients in the present study. Rapid emergence from deep sedation was also possible, with a median of 6 minutes after discontinuation of sevoflurane administration.

There are some limitations to our study. First, we used probit regression analysis to calculate EC_95_ with a small amount of data obtained using Dixon’s up-and-down sequential method. Although its utility in calculating EC_95_ has been accepted in anesthesia research,^[23,24]^ further research is warranted to investigate the EC_95_ in a larger number of patients. Second, in typical human studies to determine MAC, anesthesia is induced and maintained at a predetermined end-tidal anesthetic concentration for 15 minutes to allow equilibration of alveolar and brain partial pressure, and a standard noxious stimulus is then applied for observing movement.^[25]^ However, when using simulation of sevoflurane transmission to the target organ (brain), it takes 9 to 12 minutes to reach a steady state between the brain and inspiration when sevoflurane inspiration concentration is kept constant during the induction period and can be lowered within 6 minutes while maintaining a constant steady alveolar concentration.^[11,12]^ In the present study, we maintained the predetermined end-tidal concentration of sevoflurane for 6 minutes and then had more time for positioning patients for the procedure at constant target end-tidal sevoflurane concentration. Therefore, we expected that the brain concentration of sevoflurane would be similar to the end-tidal sevoflurane concentration before the procedure. However, we could eliminate the possibility of movement in response to a noxious stimulus at this transient stage if we were able to maintain the end-tidal concentration of sevoflurane for a longer period.

## 5. Conclusion

We found that botulinum toxin type A injection can be successfully administered, allowing spontaneous breathing in 50% of children with CP aged between 3 and 12 years at a predicted end-tidal sevoflurane concentration of 1.76 ± 0.15% with minimal adverse events according to Dixon’s up-and-down sequential allocation method. From the dose‐response curve using the probit regression analysis, the predicted EC_50_ and EC_95_ of sevoflurane without movement were 1.77% and 2.09%, respectively.

## Author contributions

**Conceptualization:** Eunhee Lee, Sung Mee Jung.

**Data curation:** Eunhee Lee, Kanghui Kim.

**Formal analysis:** Eunhee Lee, Sung Mee Jung, Kanghui Kim.

**Investigation:** Sung Mee Jung.

**Methodology:** Eunhee Lee, Sung Mee Jung, Kanghui Kim.

**Supervision:** Sung Mee Jung.

**Writing – original draft:** Eunhee Lee, Sung Mee Jung, Kanghui Kim.

**Writing – review & editing:** Kanghui Kim, Jongyoon Baek, Sung Mee Jung.
